# Current Approaches to the Management of Sentinel Node Procedures in Early Vulvar Cancer in Germany: A Web-Based Nationwide Analysis of Practices

**DOI:** 10.3390/jcm12052048

**Published:** 2023-03-04

**Authors:** Roxana Schwab, Kathrin Stewen, Theresa-Louise Bührer, Mona W. Schmidt, Josche van der Ven, Katharina Anic, Valerie C. Linz, Bashar Haj Hamoud, Walburgis Brenner, Katharina Peters, Anne-Sophie Heimes, Katrin Almstedt, Slavomir Krajnak, Wolfgang Weikel, Marco J. Battista, Christian Dannecker, Annette Hasenburg

**Affiliations:** 1 Department of Gynecology and Obstetrics, University Medical Center Mainz, Langenbeckstraße 1, 55131 Mainz, Germany; 2 Department for Gynecology, Obstetrics and Reproductive Medicine, Saarland University Hospital, 66421 Homburg, Germany; 3 Department of Gynecology and Obstetrics, University Medical Center Augsburg, Stenglinstr. 2, 86156 Augsburg, Germany

**Keywords:** vulvar cancer, rare disease, sentinel node biopsy, guidelines, certified oncological center

## Abstract

Background: Lymph node involvement is the most important prognostic factor for recurrence and survival in vulvar cancer. Sentinel node (SN) procedure can be offered in well-selected patients with early vulvar cancer. This study aimed to assess current management practices with respect to the sentinel node procedure in women with early vulvar cancer in Germany. Methods: A Web-based survey was conducted. Questionnaires were e-mailed to 612 gynecology departments. Data were summarized as frequencies and analyzed using the chi-square test. Results: A total of 222 hospitals (36.27%) responded to the invitation to participate. Among the responders, 9.5% did not offer the SN procedure. However, 79.5% evaluated SNs by ultrastaging. In vulvar cancer of the midline with unilateral localized positive SN, 49.1% and 48.6% of respondents, respectively, would perform ipsilateral or bilateral inguinal lymph node dissection. Repeat SN procedure was performed by 16.2% of respondents. For isolated tumor cells (ITCs) or micrometastases, 28.1% and 60.5% of respondents, respectively, would perform inguinal lymph node dissection, whereas 19.3% and 23.8%, respectively, would opt for radiation without further surgical intervention. Notably, 50.9% of respondents would not initiate any further therapy and 15.1% would opt for expectant management. Conclusions: The majority of German hospitals implement the SN procedure. However, only 79.5% of respondents performed ultrastaging and only 28.1% were aware that ITC may affect survival in vulvar cancer. There is a need to ensure that the management of vulvar cancer follows the latest recommendations and clinical evidence. Deviations from state-of-the-art management should only be after a detailed discussion with the concerned patient.

## 1. Introduction

Vulvar cancer is a rare cancer affecting elderly patients, with a reported worldwide incidence of 4.6 per 100,000 women per year. In Germany, 3301 new cases were reported in 2017 [1]. The incidence has been rising over the last few decades, with a 20% increase observed between 1973 and 2000 [2]. It is also occurring at younger ages; the mean age at diagnosis has decreased from 69 years to 55 years between 1979 and 1993 [3].

Vulvar cancer spreads primarily via the lymphatics. Lymph node involvement is the most important predictor of recurrence and survival and therefore a key factor to be considered when selecting the treatment approach [4,5,6]. Assessment of the lymphatic drainage is an integral part of surgical procedures for vulvar cancers of stage IB and above. However, only 25–35% of patients with early-stage disease are diagnosed with lymph node metastases [4,5,7,8,9,10], meaning that up to 75% of patients unnecessarily undergo inguinal lymph node dissection (IL) and suffer adverse outcomes, such as lymphocele and chronic lymphedema of the lower limb [10,11,12,13,14].

The sentinel node (SN) procedure can be offered as a treatment procedure in defined clinical settings and well-selected patients, such as clinically node-negative disease and in stage T1B tumors with size < 4 cm [10,15,16,17,18,19]. A survey performed in 2016 in Germany revealed that 73% of the hospitals offered the SN procedure [20]. SN dissection aims to identify the single node most likely to harbor subclinical disease and, thus, achieve oncological accuracy and safety while also minimizing short-term and long-term morbidity.

Van der Zee reported a false-negative rate of 2.3% for SN biopsy and a 3-year disease-specific survival rate of 97% following negative SN biopsy in patients with unifocal vulvar disease [8]. The largest prospective clinical trial to date—which included 452 women undergoing SN procedure and, subsequently, additional lymphadenectomy—showed that the SN procedure had a sensitivity of 91.7% per patient and false-negative predictive value of 2% in patients with tumors smaller than 4 cm [10].

However, in some circumstances, the accuracy of SN biopsy remains controversial [17]. Previous surgery of the vulva or the inguinal region may negatively impact the accuracy of SN biopsy. In patients with carcinoma involving the midline and unilaterally positive SN, as well as in patients with ITCs or micrometastases, the value of the SN procedure is not well studied and current data are inconclusive [18,21].

Moreover, national and international guidelines may slightly differ in the recommendation for managing the SN procedure in early cancer of the vulva (Table 1).

The aim of this study was to assess the adherence to current findings for the management of early vulvar cancer in Germany, especially with regard to the SN biopsy procedure, and to examine the clinical management adopted in specific situations. The ultimate goal is to improve and standardize the management of vulvar cancer in the country.

## 2. Materials and Methods

### 2.1. Development and Web Administration of the Questionnaire

A Web-based questionnaire was developed to assess the approach adopted for the management of lymph node involvement in vulvar cancer in German hospitals on the basis of the current literature (Supplementary Material). The questionnaire was validated by the experts of the Gynecological Oncology Working Group (AGO) for vulvar cancer, representing the German experts in the respective medical field. In this study, we report the results related to the use of SN mapping and the current diagnostic and surgical approaches adopted for the management of early vulvar cancer.

A direct link to the Web-based questionnaire was sent to all German hospitals having a gynecology department. The Web site included information regarding the aim of the study. If there was no response to the first e-mail invitation to participate in the survey, three follow-up e-mails were sent. The survey link was active from 14 February to 12 March 2021. The questionnaire took about 15 min to complete and was to be answered only once. All questionnaires were included in the final analysis, even if not all questions had been answered; therefore, the total number of respondents varied for each question, and the reported percentages are the proportion of respondents to each question.

### 2.2. Statistical Analysis

Data were analyzed using SPSS 26.0 (IBM Corp., Armonk, NY, USA). Data were summarized as means (± standard deviation) or absolute frequencies and proportions (%) as appropriate. The chi-square test was used to assess the significance of differences between proportions. Bivariate logistic regression analyses with the independent variables “being a certified gyneco-oncological center”, working in a “department ≥ 10 patients with primary cancer of the vulva per year”, and “surgical experience ≥ 10 years” were applied to determine the odds for adherence to current guidelines. Statistical significance was at *p* < 0.05. The McNemar test was used to determine significant differences in dichotomous dependent variables between two related groups.

## 3. Results

### 3.1. Demographic Characteristics of the Study Sample

Invitation to participate in the survey was sent to 612 hospitals. The questionnaire was accessed (“clicks”) by 222 hospitals/gynecologists; of these, 215 hospitals/gynecologists either fully or partially filled in the questionnaire. The demographic characteristics of the study group are displayed in Table 2.

While 90.5% of respondents considered the SN procedure as an option in patients with early-stage vulvar cancer (Table 2), 9.5% did not offer the SN procedure to patients. About 50.2% of hospitals reported to treat < 10 patients with primary cancer of the vulva per year, and 78.3% of them were non-certified gyneco-oncological centers (χ^2^ = 36.640; *p* < 0.001). The hospitals that reported to treat ≥ 10 patients with recurrent cancer of the vulva per year were 84% certified gyneco-oncological centers (χ^2^ = 20.337; *p* < 0.001), and all had a gynecological oncologist (*p* < 0.001).

The most frequent reasons for not offering the SN procedure were the following (multiple answers possible): a small number of vulvar cancer patients seen per year (47.4%); the belief that, from the oncological perspective, IL is at least as safe as the SN procedure (47.1%); lack of experience with the SN procedure in patients with vulvar cancer (42.1%); concerns regarding oncological safety (29.4%); and no equipment for ^99m^Tc detection (26.3%). Notably, 31% of respondents had no administrative or medical reasons for not performing the SN procedure in vulvar cancer patients.

Demographic characteristics in the perspective of adherence to current national and international guidelines are displayed in Table 3. The odds to perform the SN procedure were significantly higher in certified oncological centers than in non-certified hospitals (OR 16.743; 95% CI 2.197–127.603; *p* = 0.007) or in hospitals treating 10 or more patients with primary cancer of the vulva per year (Table 3).

### 3.2. Reported Benefits and Limitations/Contraindications of the SN Procedure

Of the 215 respondents, 89.2% answered the questions related to benefits and/or contraindications of the SN procedure (multiple answers possible). The majority (97%) recognized that quality of life was less affected by SN procedure than by IL. The other recognized benefits were the feasibility of excision of the lymph node with the highest probability of recurrence (54% of respondents), relatively little morbidity (14.6%), and the possibility of individualized therapy (3%), such as wishes of the patients or consideration of preexisting comorbidities. Meanwhile, 1% of respondents could not point out any benefits of the SN procedure in patients with vulvar cancer. There were no significant differences with regard to benefit acknowledgements between certified and non-certified oncological centers.

**Figure 1:**
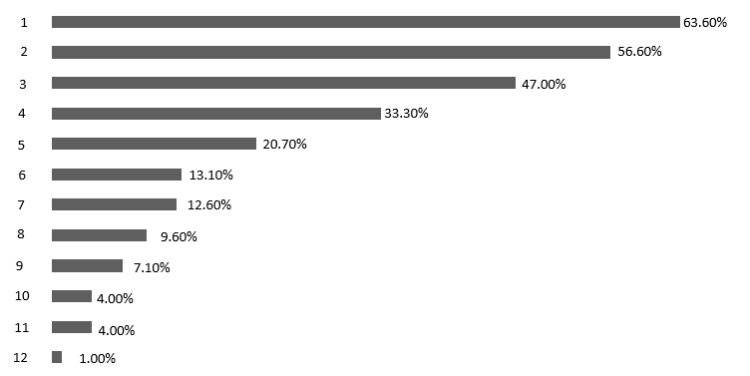
Reported contraindications to the sentinel lymphadenectomy in vulvar cancer. 1 = tumor > 4 cm; 2 = previous radiotherapy in the area of the lymphatic drainage; 3 = multifocal tumor; 4 = previous surgery of the groins; 5 = previous excisions of the vulva; 6 = no contraindications; 7 = tumor of the midline; 8 = tumor > 2 cm; 9 = suspicious lymph nodes (clinical examination and/or after imaging procedures); 10 = preexisting chronic conditions of the vulva; 11 = previous chemotherapy; 12 = individual factors.

The most frequently declared contraindication to the SN procedure in vulvar cancer (multiple answers possible) was tumor diameter > 4 cm (63.6%), followed by previous radiotherapy of the groin (56.6%), and multifocality (47%); meanwhile, 13.1% of respondents were not aware of any contraindications (Figure 1). The odds of not being aware of any contraindication regarding the SN procedure in vulvar cancer were significantly lower in certified oncological centers (OR 0.210; 95% CI 0.070–0.635; *p* = 0.006) or in centers treating ≥ 10 patients with primary cancer of the vulva per year (OR 0.392; 95% CI 0.162–0.950; *p* = 0.038) (Table 3), while acknowledging the tumor diameter of > 4 cm as a contraindication of the above-mentioned procedure was significantly higher in certified centers than in non-certified hospitals (OR 2.761; 95% CI 1.510–5.048; *p* = 0.001) (Table 3). The odds of recognizing previous surgery of the vulva or of the groins as contraindications were significantly increased in those with surgical experience of ≥ 10 years (Table 3).

### 3.3. Management of the Standard SN Procedure

The most frequently used tracer (multiple answers possible) for the SN procedure was technetium-99 m (^99m^Tc; 94.4% of respondents), followed by Patent Blue (30.5%), indocyanine green (ICG; 23.7%), methylene blue (8.5%), and Sentimag^®^ (3.40%). The combination of ^99m^Tc and one of the other tracers was used by 50.3% of respondents; the most frequently selected combination was ^99m^Tc and Patent Blue (27.93%). The staining with ICG was significantly mere often performed in certified hospitals than in non-certified centers (OR 2.148; 95% CI 1.058–4.364; *p* = 0.034) (Table 3), while no differences were observed with respect to the other tracer.

According to 68.8% of respondents, intraoperative frozen section was used for the examination of the SN. With respect to the histopathological processing of the SN, 31.3% of respondents stated that the SN was processed by hematoxylin and eosin (HE) staining. When HE staining showed no cells suspicious for malignancy, 79.5% of respondents stated that they would perform ultrastaging, and 11.9% were not performing this method, while the rest was not aware of the method used by the respective pathologist. The ultrastaging procedure was performed significantly more often in certified oncological centers than in non-certified hospitals (OR 2.450; 95% CI 1.150–5.224; *p* = 0.020), as well as hospitals treating ≥ 10 patients with primary cancer of the vulva per year (OR 2.686; 95% CI 1.268–5.687; *p* = 0.010) (Table 3).

### 3.4. Management of the SN Procedure in Specific Situations

When a metastatic lymph node was suspected prior to the SN procedure, 54.8% of respondents would perform preoperative biopsy of the suspicious node, but 37.9% would opt for upfront IL and 0.6% for upfront radiation therapy; meanwhile, 6.80% would continue with the scheduled SN procedure.

After detecting a localized positive SN in patients with vulvar cancer of the midline, 48.6% of respondents would perform bilateral IL, 49.1% would prefer a systematic dissection of the ipsilateral lymph nodes, and 2.3% would not perform any further surgical procedures. Regarding the management after previous vulvar surgery (second tumor of the vulva), 82.7% of respondents stated that they would perform the SN procedure. Notably, only 16.2% of respondents would offer a repeat SN procedure.

**Figure 2:**
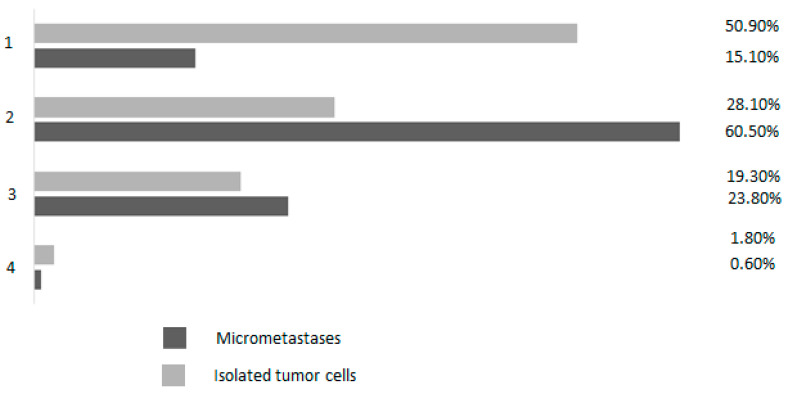
Management after detection of micrometastases (black bars) and management after detection of isolated tumor cells in sentinel nodes (grey bars) in the percentage of respondents. 1 = no further procedures, as the prognostic relevance is unclear; 2 = ipsilateral IL; 3 = ipsilateral radiation without surgical intervention; 4 = individualized procedure.

If ITCs were detected, 50.9% of respondents would not initiate any further procedures, as they assumed that ITCs in the groin lymph nodes of patients with vulvar cancer had no prognostic relevance. However, 28.1% would opt for ipsilateral IL and 19.3% would advise ipsilateral radiation without further surgical intervention (Figure 2). In patients with micrometastases, 15.1% of participants would not initiate any further procedure, 60.50% would perform ipsilateral IL, and 23.8% would offer ipsilateral radiation without further surgical intervention (Figure 2).

Regarding management after previous excision of the vulva, 82.7% of respondents would offer the SN procedure, while 17.3% of respondents would not.

With respect to a repeat SN procedure (SN procedure after a previous SN procedure), 16.2% of respondents would offer this procedure in vulvar cancer patients, while 83.8% would not. The reasons offered for avoiding a repeat SN procedure in vulvar cancer included insufficient evidence regarding the long-term outcome and oncological safety (82.5%), the belief that it was still an experimental procedure (56.1%), and the presence of atypical lymphatic drainage after a previous SN procedure (46.6%). Only 26 respondents answered the question on intraoperative experience with a repeat SN procedure; while 61.5% stated that the surgical procedure was more complex, 42.3% stated that the number of retrieved LN was lower than in a standard first SN procedure.

### 3.5. Imaging Procedures in a Standard SN Procedure and in Specific Situations

Practices related to imaging prior to the SN procedure were also assessed. For a standard SN procedure, 87% of respondents would perform ultrasound of the groins; the other favored modalities were lymphoscintigraphy (48%), computed tomography (CT; 43.5%), magnetic resonance imaging (MRI; 29.4%), single-photon emission computed tomography (2.3%) scan, and positron emission tomography/computed tomography scan (1.1%). Meanwhile, 1.7% would not perform any imaging procedure prior to a standard SN procedure. Lymphoscintigraphy before a SN procedure was significantly more often performed in certified than in non-certified hospitals (OR 1.823; 95% CI 1.007–3.330; *p* = 0.048), while MRI was significantly rarely performed in certified centers (OR 0.291; 95% CI 0.143–0.589; *p* = 0.001).

The use of imaging was largely the same for repeat SN procedures and for SN procedures after excision of the vulva. However, lymphoscintigraphy was significantly less often performed prior to the SN procedure after excision of the vulva (*p* = 0.001).

## 4. Discussion

In 2008, the first report of the GROningen International Study on Sentinel nodes in Vulvar cancer (GROINSS-V-I) study showed that the SN procedure was non-inferior to IL with respect to oncological safety in patients with vulvar cancer, but clearly superior with respect to long-term morbidity [8]. Since then, the proportion of patients receiving the SN procedure in Germany has steadily increased from 11.4% during 1998–2008 to 39.1% during 2009–2013 [22] and to 73% in 2016 [20]. The present survey showed that, in 2021, more than 90% of German hospitals treating patients with vulvar cancer applied the SN procedure when indicated. Half of the respondents in our survey were treating < 10 patients with vulvar cancer per year, and significantly more of these hospitals were non-certified gyneco-oncological centers. Moreover, approximately 15% of respondents stated that they do not provide treatment by a gynecological oncologist in the respective institution. A previous study has shown that the false-negative rate of the SN procedure can be as high as 27% in the hands of surgeons unfamiliar with the procedure [23]. In our survey, working in a low-volume center and unfamiliarity with the procedure were the reasons offered by almost half of those who stated that they would not offer the SN procedure to patients with vulvar cancer. In contrast, working in a certified oncological center significantly increased the odds of offering the SN procedure in women with early vulvar cancer. Because the SN procedure is technically challenging, several experts recommend that the procedure be performed only in high-volume oncological centers with a minimum of 5–10 SN procedures per surgeon per year. As vulvar cancer has a low incidence, centralization of treatment should be considered [8,24,25].

Ultrastaging is an enhanced pathological analysis in which serial node sections are stained by immunohistochemical procedures to detect ITCs or micrometastases [26,27]. Ultrastaging can increase the sensitivity and specificity of the SN procedure by detecting more true-positive nodes and reducing false-negative results [8,10,27,28,29]. Only 79.5% of respondents in our survey were aware that ultrastaging was performed in patients with vulvar cancer when HE staining of the SN shows no signs of malignancy; this could be due to ignorance regarding the importance of conducting the costly and time-consuming ultrastaging or due to poor communication between German gynecologists and pathologists. Ultrastaging is crucial in patients with vulvar cancer, as up to 41.7% of cases of ITCs or micrometastases are detected only by this procedure [8,10,28,30]. Routine pathological examination detects positive nodes in only 59% of cases [30]. The GROINSS-V-I study indicated that strict adherence to the SN protocol and awareness of contraindications could prevent false-negative results [8]. According to national and international guidelines, the two most important contraindications are tumor size > 4 cm and tumor multifocality [16,17,18,19]. In our survey, only 63.6% and 47% of respondents, respectively, recognized tumor size > 4 cm and multifocality as contraindications. Levenback et al. found that the false-negative rate of the SN procedure was 2% in women with tumors < 4 cm in size but 7.4% in women with tumors 4–6 cm in size, suggesting the possibility of fatal undertreatment of the groins in those with large tumors [10]. In the GROINSS-V-I study, 252 (62.5%) women had tumors of the midline but showed no impairment in long-term outcome; however, two women with multifocal disease developed tumor recurrence within a short period of time [8]. Subsequent analyses revealed that, in multifocal disease, peritumoral injection of tracer may not fully reveal the extent of the tumors; thus, these patients should be treated with upfront IL [8].

Several types of tracers (^99m^Tc, blue dye, and Patent Blue) can help visualize the SN. National and international guidelines mandate the use of a radioactive tracer, as metastases are unlikely to be missed with this technique [15,18]. Our survey showed that most German institutions follow these guidelines (94.4% of respondents affirmed the use of ^99m^Tc colloid). In a previous study, the use of blue dye identified only 82.5% of positive nodes [26]. Other studies have shown that only 56% of radioactive isotope–traced nodes are stained by blue dye [9,31]. Oonk et al. found that the SN procedure performed using the combination of a radioactive tracer and another tracer had a negative predictive value of nearly 100% [9,24]. In our survey, only half of the respondents confirmed the use of the double-tracer method; nevertheless, neither the national nor the international guidelines recommended the use of the double-tracer method as mandatory [15,16,17,18,19]. In recent years, new tracing methods have been introduced. Although only 6.8% of German hospitals used ICG in 2016 [20], 23.7% of our respondents reported having experience with ICG tracing. According to some authors, ICG tracing of groin lymph nodes in patients with vulvar cancer provides a sensitivity of nearly 100% and a false-negative rate of 0% [32,33]; thus, it may be an excellent option for centers with experience with this technique. Nevertheless, there are no reports regarding long-term outcomes after ICG, so the performance should be restricted to experienced teams.

For the treatment of recurrent disease, IL is recommended if it has not already been performed [15]. Data on the accuracy and safety of a repeat SN procedure in vulvar cancer are scarce, and, consequently, only 16.2% of respondents in our survey said that they would offer the SN procedure to their patients. In a retrospective study of five oncological centers in the Netherlands, van Doorn et al. showed that a repeat SN procedure is feasible, though more challenging than a primary SN node biopsy [34]. In their study, no groin recurrence occurred during the follow-up period of 27 months [34]. A repeat SN procedure might be an option in older patients or patients who wish to minimize short-term and long-term morbidity. However, as a matter of course, thorough counseling regarding the risks and benefits of therapy should precede every deviation from state-of-the-art treatment.

In our survey, only 48.6% of respondents said that they would perform bilateral IL in patients with vulvar cancer of the midline and unilateral positive SN. According to current evidence, bilateral IL should be performed in patients with unilateral positive SN. In a recent study on women presenting with midline lesions, an SN positivity rate of 22.2% on the contralateral side was described (median depth of invasion is 8.5 mm in those with contralateral metastases), indicating that understaging of the groins might be fatal [35]. Deeper invasion is associated with an increased risk of metastatic disease and so, for women who are concerned about possible long-term effects of bilateral IL, depth of invasion should be taken into account during counseling [19,35].

A proportion of 82.7% of respondents would offer the SN procedure to women with previous excision of the vulva; however, 20.7% of respondents listed previous excision of the vulva as a relative contraindication for the SN procedure. Crosbie et al. hypothesized that vulvar resection would result in damage to lymphatics and altered lymph flow and could thus affect the accuracy of the SN procedure [36]. In their study, significantly fewer SNs were detected in the 15 patients with previous excision of the vulva than in those without surgery of the vulva (*n* = 17). However, the recurrence rate was not significantly different between the two groups after a follow-up of 62 months [36]. In recent studies, the SN detection rate in operated patients with scar injection (87.5–100%) and the disease-free survival rates were similar to those in patients without previous vulvar tumor excision [10,37,38].

In contrast to breast cancer patients, in whom the presence of small metastases (<2 mm) in the SN do not impact survival (presumably due to the specific biology of breast cancer or the frequently applied additional adjuvant treatment), patients with vulvar cancer appear to have poorer oncological outcomes if no further interventions are undertaken. In a previous study, non-SN metastases were detected in 4.2% of patients with ITCs in the SN and in 10.5% of patients with micrometastases [30]. The possibility of non-SN metastases increased with the diameter of the SN metastasis, but the authors did not determine a cutoff size that could accurately predict the absence of non-SN metastases [30]. The 5-year survival was 97% in patients with ITCs and 88% in patients with metastases ≤ 2 mm [30]. The authors of the GROINSS-V-I study concluded that the presence of SN metastasis, irrespective of size, was an indication for further groin treatment, with surgery being the first choice for ensuring locoregional control [30]. It is therefore alarming that 50.90% and 15.1% of respondents in our survey would not initiate any further treatment after the detection of ITCs or micrometastases, respectively. Our data suggest that, currently, not all German patients with vulvar cancer will benefit from state-of-the-art treatment and might therefore lose years of potential life. Nevertheless, in our survey, for patients with ITCs or with micrometastases, 19.30% and 23.80% of respondents, respectively, said that they would advise ipsilateral radiation without further surgical intervention. This is reassuring, because 2-year follow-up data from the GROINSS-V-II study suggest that radiotherapy of the groins with 50 Gy was safe in patients with ITCs or micrometastases, as the isolated groin recurrence rate was 1.6% [39,40].

Accurate preoperative staging is crucial for deciding the extent of groin surgery and for selecting patients suitable for the SN procedure, thus avoiding unnecessary groin dissection and related morbidity and also increasing oncological safety in women eligible for the SN procedure. In this survey, 3.2% of respondents stated that they do not perform any imaging procedure prior to surgery of the groins, thus not following national or international standards of treatment. Imaging procedures are particularly important when planning surgical management in patients with vulvar cancer, as clinical assessment of inguinal nodes is often inaccurate—with a false-negative rate of 16–24% and a false-positive rate of 24–41% [41,42].

Up to 88% of respondents in our survey would perform preoperative ultrasound of the groins in patients with vulvar cancer and 54.8% would additionally assess suspicious lymph nodes by cytological or histological methods to determine the optimal surgical procedure. The combination of preoperative ultrasound and fine-needle aspiration cytology has a sensitivity of 93% and a specificity of 100% [43].

Lymphoscintigraphy is a non-invasive, well-established method for evaluating the anatomical pattern of lymphatic distribution [44], especially in specific situations [45], such as a previous SN procedure. In our survey, 58.1% of respondents used this imaging procedure, especially those working in certified cancer centers.

Other imaging procedures are less sensitive in this population. However, in our survey, 48.8% and 33.30% of respondents, respectively, stated that they usually performed CT or MRI scan prior to surgery for vulvar cancer, while preoperative CT did not modify the initial treatment strategy in a previous study [46]. PET scan also appears to have a relatively low value for predicting lymph node metastasis in women with vulvar cancer [47]. MRI has no value for preoperative assessment of lymph node involvement in vulvar cancer, with sensitivity, specificity, positive predictive value, and negative predictive value of only 52%, 85%, 46%, and 87%, respectively [48].

### Limitations

An obvious limitation of our study is the design; this was an online survey using an invitation e-mail. With multiple reminders sent to non-respondents, we achieved a final response rate of 36.23%, which is comparable to the response rates achieved in previous e-mail-based studies [49,50]. In surveys, there is a potential risk for bias if non-respondents differ significantly from respondents with respect to demographic and practice variables [51]. However, non-response bias may be of less concern in surveys conducted among physicians, as they constitute a relatively homogeneous population with regard to knowledge, training, and attitudes [52]. Previous studies have shown that higher response rates are not associated with lower response bias [51].

## 5. Conclusions

There is high diversity in the management of vulvar cancer in Germany despite the availability of specific guidelines that are updated regularly. Although vulvar cancer is a rare disease, treatment in Germany is not centralized. We showed that centralization by treating women with vulvar cancer in certified gyneco-oncological centers led to increased compliance regarding following the current guidelines. It is crucial to ensure adherence to the latest evidence and current guidelines regarding upfront treatment for patients with vulvar cancer. In order to improve the care for patients with malignant diseases, it is necessary to implement regular internal and external monitoring of treatment procedures, continuing education, as well as centralization. The effect of such interventions should be checked by subsequent surveys.

## Figures and Tables

**Table 1:** Comparison of recommended procedures with regard to sentinel node procedure by different guidelines.

	AWMF Guideline [18]	ESGO Guideline [16]	NCCN Guideline [17]
SN recommended, if feasible			
	+	+	+
Prerequisite for SN procedure in early-stage vulvar cancer			
Preoperative assessment	Clinical examination and sonographic imaging of the groins are required	Clinical examination and imaging of the groins (any modality) are required	Clinically/radiologically negative groin nodes
Tumor < 4 cm	+	+	+
Unifocallity of the tumor	+	+	+
Tracer	Use of radioactive tracer is mandatory, and use of blue dye is optional	Use of radioactive tracer is mandatory, and use of blue dye is optional	Dual tracer recommended (i.e., radiocolloid and dye)
Ultrastaging, if HE staining is negative	+	+	+
Expertise of the surgical team	Surgical team/surgeon with expertise in the SN procedure	At least 5 to 10 patients with the SN procedure per year per surgeon	SN procedure by high-volume SN surgeon
SN procedure in specific situations			
SN procedure after history of previous vulvar surgery	ns	ns	-
SN procedure after a previous SN procedure	ns	ns	ns
Bilateral IL after unilateral positive SN and contralateral negative SN in tumor of the midline	No sufficient data	Contralateral inguinofemoral lymphadenectomy may be performed when ipsilateral nodes show metastatic disease	ns
Procedure after detection of isolated tumor cells or micrometastases in the SN	No sufficient data	Any size of metastatic disease is identified in the sentinel lymph node: inguinofemoral lymphadenectomy in the groin with the metastatic sentinel lymph node should be performed	Increased risk for non-SN metastases, if SLN is positive, completion lymphadenectomy or treatment of the affected groin may be warranted

AWMF, Arbeitsgemeinschaft der Wissenschaftlichen Medizinischen Fachgesellschaften e.V.; ESGO, European Society of Gynecologic Oncology; NCCN, National Comprehensive Cancer Network; SN, sentinel node; IL, inguinal lymph node dissection; HE, hematoxylin and eosin; +, recommended; -, not recommended; ns, not specified.

**Table 2:** Demographic characteristics of study participants.

	% (*n*/N)
Hospitals	
University hospital	12.1 (26/215)
Teaching hospital	55.3 (119/215)
Hospital offering maximal care	7.9 (17/215)
General hospital	24.7 (53/215)
Certified gyneco-oncological center	
Yes	42.3 (91/215)
No	67.7 (124/215)
Surgical experience in the field of gyneco-oncology	
<10 years	16.3 (35/215)
10–15 years	19.5 (42/215)
≥15 years	64.2 (138/215)
Number of oncological specialists employed	
None	15.5 (33/213)
1	30.0 (64/213)
2	31.5 (67/213)
>2	23.0 (49/213)
Number of patients with primary vulvar cancer/year/hospital	
<10	50.2 (106/211)
≥10	49.8 (105/211)
Number of patients with recurrent vulvar cancer/year/hospital	
<10	88.2 (186/211)
≥10	11.8 (25/211)
Sentinel node biopsy procedure	
No	9.5 (20/210)
Yes	90.5 (190/210)

N = total number of hospitals providing data to the question; *n* = number of respondents to the specific answer.

**Table 3:** Demographic characteristics in the perspective of adherence to current national and international guidelines.

	Independent Variable	Being a Certified Gyneco-Oncological Center (co: Center Not Certified)	Department with ≥10 Patients with Primary Vulvar Cancer/Year (co: <10 Patients/Year)	Surgical Experience ≥10 Years (co: <10 Years of Experience)
Dependent Variable				
Provide SN procedure				
Hospitals offering SN procedure		**OR 16.743; 95% CI 2.197–127.603; *p* = 0.007**	**OR 4.545; 95% CI1.465–14.106; *p* = 0.009**	OR 0.905; 95% CI 0.250–3.276; *p* = 0.879
Benefits of SN procedure				
Excision of the lymph node with the highest probability of recurrence		OR 1.136; 95% CI 0.652–1.979; *p* = 0.653	OR 1.194; 95% CI 0.688–2.071; *p* = 0.529	OR 1.197; 95% CI 0.557–2.571; *p* = 0.645
Improved quality of life		OR 1.193; 95% CI 0.376–3.780; *p* = 0.765	OR 3.626; 95% CI 0.968–13.592; *p* = 0.056	OR 1.022; 95% CI 0.215–4.851; *p* = 0.978
Less morbidity		OR 2.145; 95% CI 0.966–4.766; *p* = 0.061	OR 1.071; 95% CI 0.488–2.353; *p* = 0.864	**OR 0.317; 95% CI 0.128–0.784; *p* = 0.013**
Contraindications of SN procedure				
Tumor > 4 cm		**OR 2.761; 95% CI 1.510–5.048; *p* = 0.001**	OR 1.680; 95% CI 0.949–2.974; *p* = 0.075	**OR 0.260; 95% CI 0.095–0.709; *p* = 0.008**
Multifocal tumor		OR 1.695; 95% CI 0.969–2.966; *p* = 0.065	OR 0.944; 95% Ci 0.543–1.639; *p* = 0.837	OR 0.547; 95% CI 0.252–1.186; *p* = 0.127
Suspicious lymph nodes (clinical examination and/or after imaging procedures)		OR 0.420; 95% CI 0.158–1.718; *p* = 0.284	OR 0.599; 95% CI 0.189–1.898; *p* = 0.384	OR 2.422; 95% CI 0.305–19.215; *p* = 0.402
Previous surgery of the vulva		OR 1.078; 95% CI 0.540–2.149; *p* = 0.832	OR 0.576; 95% CI 0.414–1.634; *p* = 0.576	**OR 8.955; 95% C 1.184–67.738; *p* = 0.034**
Previous surgery of the groins		OR 0.757; 95% CI 0.416–1.379; *p* = 0.363	OR 0.687; 95% CI 0.381–1.240; *p* = 0.213	**OR 2.807; 95% CI 1.026–7.680; *p* = 0.044**
No contraindications		**OR 0.210; 95% CI 0.070–0.635; *p* = 0.006**	**OR 0.392; 95% CI 0.162–0.950; *p* = 0.038**	OR 5.034; 95% CI 0.657–38.591; *p* = 0.120
Diagnostic workup previous to SN procedure				
No previous imaging procedure		OR 2.241; 95% CI 0.200–25.167; *p* = 0.513	OR 1.783; 95% CI 0.159–20.024; *p* = 0.639	OR 0.362; 95% CI 0.032–4.138; *p* = 0.414
Ultrasound of the groins		OR 1.065; 95% CI0.462–2.451; *p* = 0.883	OR 2.006; 95% CI 0.855–4.707; *p* = 0.110	OR 1.346; 95% CI 0.461–3.929; *p* = 1.346
Lymphoscintigraphy		**OR 1.823; 95% CI 1.007–3.300; *p* = 0.048**	OR 1.036; 95% CI 0.574–1.871; *p* = 0.906	OR 1.485; 95% CI 0.652–3.382; *p* = 0.346
Magnetic resonance imaging (MRI)		**OR 0.291; 95% CI 0.143–0.589; *p* = 0.001**	OR 0.603; 95% CI 0.314–1.157; *p* = 0.128	OR 1.1606; 95% CI 0.611–4.224; *p* = 0.337
Computed tomography (CT scan)		OR 0.657; 95% CI 0.362–1.194; *p* = 0.168	OR 0.689; 95% CI 0.384–1.268; *p* = 0.238	OR 0.716; 95% CI 0.319–1.607; *p* = 0.418
Tracer				
^99m^Tc		OR 1.884; 95% CI 0.546–6.496; *p* = 0.316	OR 1.639; 95%CI 0.500–5.377; *p* = 0.415	OR 1.085; 95% CI 0.225–5.238; *p* = 0.919
Methylene blue dye		OR 1.291; 95% CI 0.447–3.726; *p* = 0.636	OR 0.462; 95% CI 0.148–1.438; *p* = 0.183	OR 0.719; 95% CI 0.189–2.733; *p* = 0.629
Patent blue V dye		OR 1.039; 95% CI 0.548–1.968; *p* = 0.907	OR 1.597; 95% CI 0.833–3.064; *p* = 0.159	OR 1.095; 95% CI 0.450–2.667; *p* = 0.841
Indocyanine green (ICG)		**OR 2.148; 95% CI 1.058–4.364; *p* = 0.034**	OR 1.986; 95% CI 0.959–4.112; *p* = 0.065	**OR 0.274; 95% CI 0.118–0.639; *p* = 0.003**
SN workup				
Performing intraoperative frozen section		OR 0.610; 95% CI 0.307–1.214; *p* = 0.159	OR 0.711; 95% CI 0.358–1.414; *p* = 0.331	OR 1.433; 95% 0.531–3.868; *p* = 0.477
Ultrastaging of the SN		**OR 2.451; 95% CI 1.150–5.224; *p* = 0.020**	**OR 2.686; 95% CI 1.268–5.687; *p* = 0.010**	OR 1.548; 95% CI 0.623–3.847; *p* = 0.346

SN, sentinel node; co, controls; HE, hematoxylin and eosin; OR, odds ratio; CI, confidence interval. Significant values are denoted in bold, as statistical significance was at *p* < 0.05.

## Data Availability

All relevant data were included in the manuscript.

## Supplementary Materials

The following supporting information can be downloaded at https://www.mdpi.com/article/10.3390/jcm12052048/s1.
