# Supplementary material for: Current Approaches to the Management of Sentinel Node Procedures in Early Vulvar Cancer in Germany: A Web-Based Nationwide Analysis of Practices
Source: J Clin Med. 2023 Mar 4;12(5):2048. doi: 10.3390/jcm12052048 (PMC10004173; doi:10.3390/jcm12052048)
Supplement: Supplementary file 1 [file jcm-12-02048-s001.zip › jcm-2184932-supplementary.pdf]

Questionnaire SN procedure in early cancer of the vulva

Your hospital is a

University hospital

Teaching hospital

Hospital offering maximal care

General Hospital

Your hospital is a certified gyneco-oncological center

Yes

No

How long are you working in the field of surgical gyneco-oncology

< 10 years

10-15 years

≥ 15 years

How many gynoco-oncological specialists are employed in your hospital

None

1

2

>2

How many patients with primary cancer of the vulva are treated in your hospital per year

0

1-9

10-19

≥20

How many patients with recurrent cancer of the vulva are treated in your hospital per year

0

1-9

10-19

≥20

Does your hospital provide SN procedure

No

Yes, if feasible

In your opinion, which benefits has the SN procedure compared to IL (multiple answers)

No benefits

Higher detection rate for metasatic lymph nodes

Excision of the lymph node with the highest probability of recurrence

Improved quality of life

Others:

In your opinion, which contraindications has the SN procedure (multiple answers)

No contraindications

Previous surgery of the groins

Tumor > 2 cm

Tumor > 4 cm

Multifocal tumors

Tumors of the midline

Chronic disease of the vulva

Previous radiotherapy of the groins/lymphatic drainage

Previous chemotherapy

Others:

For how long do you perform the SN procedure in your hospital

<1 y

1 to <5 y

5 to <10 y

≥ 10 y

Which diagnostic procedures do you apply previous to SN procedure (multiple answers)

None

Lymphoscintigraphy

Ultrasound of the groins

Magnetic resonance imaging (MRI)

Computed tomography (CT scan)

positron emission tomography

Others:

Which procedure do you apply, if metastatic nodes are suspected in the imaging procedures previous to the SN procedure

No regimen changes, the SN procedure will be performed

No additional diagnostic procedure, but I will perform IL

Biopsy of the suspected node, further surgical procedure dependent on the pathological results

Primary radiotherapy of the lymphatics

Which tracer do you apply (multiple answers)

Methylene blue dye

Patent blue dye

^{99m}Tc

ICG

Sentimag

Others:

Do you perform frozen section during the surgery

No

Yes

How does your pathologist process the SN

I do not know

Hematoxylin and eosin (HE) staining

Ultrastaging, if HE showed no metastatic disease

Your detect a unilateral SN metastasis in a cancer of the vulva of the midline. How do you proceed?

No additional surgical procedure

Ipsilataral (unilateral) IL

Bilateral IL

You detect isolated tumor cells (≤ 0.2 mm) during ultrastaging. How do you proceed?

No further procedures, as he prognostic significance of isolated tumor cells is not clear

Ipsilateral radiotherapy without further surgical procedures

Ipsilateral IL

Others:

You detect micrometasases (0.2 mm to 2mm) during ultrastaging. How do you proceed?

No further procedures, as he prognostic significance of micrometastases is not clear

Ipsilateral radiotherapy without further surgical procedures

Ipsilateral IL

Others:

After a surgical procedure of the vulva, you detect a vulvar cancer (>1mm). Do you recommend a SN procedure (after previous surgery)

No

Yes

Which are your concerns regarding SN procedure after previous surgery of the vulva

Method is not trustworthy

I do not have sufficient experience with SN procedure after previous surgery of the vulva

Not sufficient data regarding the oncological safety with respect to SN procedure after pervious surgery of the vulva

Others:

Which imaging procedure do you perform previous to SN procedure after previous surgery of the vulva

None

Lymphoscintigraphy

Ultrasound of the groins

Magnetic resonance imaging (MRI)

Computed tomography (CT scan)

positron emission tomography

Others:

You care for a patient who had a previous SN procedure. Would you offer a repeat SN procedure (after previous SN procedure)

No

Yes

Which are your concerns regarding SN procedure after previous SN procedure (repeat SN)

No concerns

Abberant/untypical lymphatic drainage

Procedure is still experimental

No data regarding long-term oncological outcome

Others:

For how long do you perform the repeat SN procedure

….years

Which imaging procedure do you perform previous to repeat SN procedure

None

Lymphoscintigraphy

Ultrasound of the groins

Magnetic resonance imaging (MRI)

Computed tomography (CT scan)

positron emission tomography

Others:

How is your experience with the repeat SN procedure (multiple answers)

The number of lymph nodes with tracer signal is similar or higher than in the primary procedure

The number of lymph nodes with tracer signal is lower than in the primary procedure

The surgical preparation is more complex

The complication rate during the surgery is higher

Others:
